# Clinical evaluation of Alprostadil combined with Nimodipine in treatment of Cerebral Vasospasm after Subarachnoid Hemorrhage in elderly patients

**DOI:** 10.12669/pjms.39.3.6753

**Published:** 2023

**Authors:** Sisi Liu, Ning Gan, Jing Xie, Yu Zhang, Fei Su, Tongle Jia

**Affiliations:** 1 Sisi Liu, Department of Neuroscience Critical Care Unit, Baoding No.1 Central Hospital, Baoding 071000, Hebei, China; 2 Ning Gan, Department of Neurosurgery, Baoding No.1 Central Hospital, Baoding 071000, Hebei, China; 3 Jing Xie, Department of Neurosurgery, Baoding No.1 Central Hospital, Baoding 071000, Hebei, China; 4 Yu Zhang, Department of Neurosurgery, Baoding No.1 Central Hospital, Baoding 071000, Hebei, China; 5 Fei Su, Department of Neurosurgery, Baoding No.1 Central Hospital, Baoding 071000, Hebei, China; 6 Tongle Jia, Department of Neurosurgery, Baoding No.1 Central Hospital, Baoding 071000, Hebei, China

**Keywords:** Alprostadil, Nimodipine, Subarachnoid hemorrhage, Cerebral vasospasm

## Abstract

**Objective:**

To analyze the clinical efficacy of alprostadil combined with nimodipine in the treatment of cerebral vasospasm (CVS) after subarachnoid hemorrhage (SAH) in elderly patients.

**Methods:**

This is a retrospective study. According to different treatment methods, the elderly 100 patients with CVS after SAH hospitalized in Baoding First Central Hospital from March 2020 to May 2021 were randomly divided into control group and observation group, with 50 patients in each group. The control group was treated with nimodipine, while the observation group was additionally combined with alprostadil. The levels of inflammatory factors and hemorheological indexes were measured before and after treatment. The clinical efficacy was compared and the adverse reactions were observed of the two groups.

**Results:**

The overall clinical efficacy in the observation group (95.00%) was significantly higher than that in the control group (74.00%) (*p<*0.05). After treatment, serum tumor necrosis factor-α (TNF-α), interleukin-8 (IL-8), high-sensitivity C-reactive protein (hs-CRP) and hemorheological indexes such as plasma viscosity, whole blood viscosity at high shear, whole blood viscosity at low shear, hematocrit and platelet adhesion decreased significantly compared with those before treatment (*p<*0.05), which were more obvious in the observation group (*p<*0.05). During treatment, the rate of adverse reactions in the observation group was 12.00%, and that in the control group was 8.00%, without statistically significant difference between the two groups (*p>* 0.05).

**Conclusion:**

Alprostadil combined with nimodipine is markedly effective in the treatment of CVS after SAH in elderly patients. It can effectively reduce inflammatory factor levels and improve hemorheological indexes in patients, which is conducive to the repair of neurological function.

## INTRODUCTION

Subarachnoid hemorrhage (SAH) is a very dangerous and common cerebrovascular disease, which mostly occurs in the elderly, characterized by acute onset and high mortality.^1^,^2^ Cerebral vasospasm (CVS) is one of the most common severe complications in SAH patients, with an incidence of about 70%. After complicated with CVS, SAH patients usually develop into severe cerebral ischemia. If not treated in time, it may also lead to severe disability or even death. This is because the vascular diameter of patients combined with CVS will be significantly thinner, resulting in changes in cerebral hemodynamics, which will further cause the decrease in cerebral perfusion pressure and cerebral blood flow, and finally damage the self-regulating function of cerebral vessels.^3^,^4^ With the improvement in medical level, the clinical prognosis of some SAH patients has been significantly improved.

However, the clinical results have shown that the gradual decline in system function of elderly patients will significantly affect their prognosis, and the concurrent CVS is still a difficulty during the treatment.^5^ Many researchers have devoted themselves to the research on the pathogenesis of secondary CVS in SAH patients. At present, there is no final conclusion. Therefore, specific scheme for the clinical treatment of SAH combined with CVS is lacking, and calcium antagonists are mainly used.^6^ Nimodipine is fat-soluble, easy to pass through the blood-brain barrier and can rapidly dilate cerebral vessels. It is currently the most widely used Ca^2+^ antagonist in the clinical treatment of CVS.^6^,^7^ Considering the limitations of single drug in treatment, alprostadil combined with nimodipine was used in this study to treat elderly patients with CVS after SAH, and its clinical efficacy and safety were evaluated.

## METHODS

Retrospective study was used. One hundred patients with CVS (Cerebral vasospasm) after SAH (Subarachnoid hemorrhage) meeting the inclusion criteria treated in Baoding First Central Hospital from March 2020 to May 2021 were selected and divided into control group (n = 50) and observation group (n = 50) using the simple random grouping. In the control group, there were 23 males and 27 females, with an average age of (65.41 ± 5.53) years. The observation group included 22 males and 28 females, with an average age of (65.75 ± 6.28) years. No significant differences were found in gender, course of disease or age between the two groups (*p>* 0.05). The study was approved by the Institutional Ethics Committee of Baoding No.1 Central Hospital dated (No.:[2022]083; August 03, 2022), and written informed consent was obtained from all participants.

### Inclusion criteria:

- Older than 60 years old, and meeting the diagnostic criteria for SAH issued by the American Heart Association/American Stroke Association in 2012^8^;
- Main clinical manifestations included hemiplegia, aphasia and other neurological impairment, accompanied by intracranial hypertension;
- The time from onset to treatment ≤ 72 hour, and secondary SAH was excluded by head CT;
- Patients and their families signed the informed consent.

### Exclusion criteria:

- Patients with blood diseases or tumors that may lead to SAH;
- Heart, lung, kidney and other major organ dysfunction;
- Severe basic diseases;
- Allergic to drugs used during treatment.

All the patients were treated with routine symptomatic treatment such as dehydration, anti-shock, and prevention of infection and complications. On this basis, the control group was treated with nimodipine, and the observation group was additionally treated with 2 mL alprostadil injection (Guoyao Zhunzi, H20103292; Hainan Bikai Pharmaceutical Co., Ltd.; 10 μg/2- mL) in 10 mL normal saline by slow intravenous injection, once a d. The treatment cycle of the two groups was 14 days.

Evaluation of clinical efficacy: After treatment, the clinical efficacy of the two groups was evaluated. The evaluation criteria:^9^ Remarkably effective: the cerebral blood flow velocity of CVS patients returned to normal level, the damaged neurological function was significantly repaired, the clinical symptoms and signs basically disappeared, and the self-care ability was completely restored; effective: the cerebral blood flow velocity and neurological function of the patients were partially recovered, the clinical symptoms were relieved, and the self-care ability was basically achieved; invalid: the clinical symptoms and signs of the patients were not significantly improved or even aggravated. The total effective rate = remarkably effective rate + effective rate.

Before and after treatment, serum tumor necrosis factor-α (TNF-α) and interleukin-8 (IL-8) were measured using radioimmunoassay (kit: Beijing North Institute of Biotechnology Co. Ltd.) strictly according to the instructions. Serum hypersensitive C-reactive protein (hs-CRP) content was measured using enzyme-linked immunosorbent assay with an automatic biochemical analyzer (7600, Hitachi, Japan). The kit was provided by Shanghai Xitang Biotechnique Co, Ltd.

Before and after treatment, hemorheological indexes were detected using a full-automatic blood rheometer (ZL9000, Beijing Zhongchi Weiye Technology Development Co., Ltd.), including plasma viscosity, whole blood viscosity at high shear, whole blood viscosity at low shear, hematocrit and platelet adhesion. The adverse reactions of the two groups during treatment were recorded.

### Statistical Analysis:

The obtained data were analyzed and processed using SPSS 22.0. The measurement data were expressed as *χ̅*±*S*, and analyzed with the *t* test. Quantitative data were expressed as rate (%), and analyzed by the *χ*^2^ test. *P*< 0.05 was considered as statistically significant.

## RESULTS

The data on clinical efficacy of the two groups in this study are listed in Table-I. In the observation group, 27 patients showed effective results, with the total effective rate of 95.00%. In the control group, there were 14 patients with remarkable effectiveness and 23 with effectiveness, with the total effective rate of 74.00%. The clinical efficacy of the observation group was significantly higher than that of the control group (*p<* 0.05).

**Table-I:** Comparison in clinical efficacy between two groups.

Group	N	Remarkably effective	Effective	Invalid	Total effective rate (%)
Observation group	50	18	27	5	95.00
Control group	50	14	23	13	74.00
*χ* ^2^					4.336
*p*					0.037

Before treatment, no statistically significant differences were found in the detected values between the two groups (*p>* 0.05), indicating that the data were comparable. After treatment, TNF-α, IL-8 and hs-CRP decreased significantly in the two groups (*p<* 0.05), which was more obvious in the observation group (*p<* 0.05), as seen in Table-II.

**Table-II:** Comparison in inflammatory factor levels between two groups (n = 50).

Group	TNF-α ( μg/L)		IL-8 (μg/L)		hs-CRP (mg/L)	

	Before treatment	After treatment	Before treatment	After treatment	Before treatment	After treatment
Observation group	2.58 ± 0.06	1.29 ± 0.07*	0.65 ± 0.05	0.32 ± 0.10*	27.03 ± 3.84	8.93 ± 3.15*
Control group	2.56 ± 0.08	1.57 ± 0.05*	0.66 ± 0.03	0.43 ± 0.08*	27.45 ± 3.19	12.53 ± 2.73*
*t*	1.414	15.185	1.213	6.074	0.595	6.107
*p*	0.1605	< 0.001	0.228	< 0.001	0.553	< 0.001

***Notes:*** Compared with before treatment,

* p< 0.05

Before treatment, there were no statistically significant differences in hemorheological indexes between the two groups (*p>* 0.05), indicating the data were comparable. Compared with before treatment, plasma viscosity, whole blood viscosity at high shear, whole blood viscosity at low shear, hematocrit and platelet adhesion reduced significantly in both groups after treatment. Compared with the control group, the decreases in the observation group were more obvious (*p<* 0.05), as shown in Table-III.

**Table-III:** Comparison in hemorheological indexes between two groups before and after treatment (*n* = 50, *χ̅*±*S*).

Group	Plasma viscosity (mPa·s)		Whole blood viscosity at low shear (mPa·s)		Whole blood viscosity at low shear (mPa·s)		Hematocrit (%)		Platelet adhesion (%)	

	Before treatment	After treatment	Before treatment	After treatment	Before treatment	After treatment	Before treatment	After treatment	Before treatment	After treatment
Observation group	2.13 ± 0.48	1.66 ± 0.43*	9.82 ± 1.67	7.35 ± 1.28*	7.02 ± 1.14	5.41 ± 0.74*	57.25 ± 6.27	41.84 ± 5.03*	46.38 ± 6.39	34.57 ± 5.21*
Control group	2.20 ± 0.27	1.87 ± 0.35*	9.76 ± 1.54	8.71 ± 1.36*	7.10 ± 1.21	6.38 ± 0.83*	57.41 ± 6.19	49.27 ± 4.96*	46.56 ± 6.44	38.16 ± 5.62*
*t*	0.899	2.678	0.187	5.149	0.340	6.168	0.128	7.437	0.140	3.072
*p*	0.371	0.009	0.852	< 0.001	0.734	< 0.001	0.898	< 0.001	0.889	0.003

***Notes:*** Compared with before treatment,

* p< 0.05.

In the observation group, there were six patients suffering from adverse reactions, with the adverse reaction rate of 12.00%, included gastrointestinal discomfort in two patients, rashed in three patients and blood pressure dropped in one patient. In the control group, four patients presented adverse reactions, with the adverse reaction rate of 8.00%, including gastrointestinal discomfort in one patient, blood pressure drop in drop patients, and facial flushing in one patient. Through the *χ*^2^ test, the incidences of adverse reactions showed no statistically significant differences between the two groups (*p>* 0.05). The adverse reactions of the two groups were mild and did not affect the normal medication (Table-IV).

**Table-IV:** Comparison in adverse reactions between two groups (*n* = 50).

Group	Gastrointestinal discomfort	Rashes	Blood pressure drop	Facial flushing	Incidence of adverse reactions (%)
Observation group	2	3	1	0	12.00
Control group	1	0	2	1	8.00
*χ* ^2^					0.444
*p*					0.505

## DISCUSSION

In this study, alprostadil combined with nimodipine was used to treat elderly patients with CVS after SAH, and compared with the control group treated with nimodipine alone. The results showed that the clinical efficacy of the observation group was significantly higher than that of the control group, and there were no significant differences in the incidences of adverse reactions between the two groups, indicating that the combined use of alprostadil and nimodipine had a synergistic effect and significantly improved the efficacy, with safety. A large number of studies have found that the vascular wall of patients with SAH combined with CVS was accompanied by inflammatory cell infiltration, and the inflammatory reaction is an important cause for the occurrence and development of CVS.^10^,^11^ In this study, serum inflammatory factors TNF-α, IL-8 and hs-CRP levels decreased significantly in the two groups after treatment, and these levels in the observation group were significantly lower than those in the control group, suggesting that the combination of alprostadil and nimodipine can more effectively regulate inflammatory reaction and protect brain tissue. The reason may be that alprostadil and nimodipine, two Ca^2+^ antagonists, can inhibit the release of inflammatory factors by blocking Ca^2+^ channel, and their combined application has a better effect.^12^,^13^

Continuous contraction of the intracranial artery in SAH patients indicates that they are complicated with CVS. Most CVS patients present symptomatic vasospasm, accompanied by neurological dysfunction such as hemiplegia and aphasia. The incidence of CVS will increase gradually with age, and is the highest in the elderly aged 60 years.^14^,^15^ To reduce the high disability and mortality caused by CVS, effective treatment is very necessary in the early stage of CVS.^16^ It has been shown that secondary CVS causes structural and pathological changes in blood vessels, but vasodilators with single function cannot obtain good efficacy in clinical treatment. Nimodipine, a Ca^2+^ antagonist, can effectively reduce Ca^2+^ concentration in smooth muscle cells and relax spasmodic vessels. Currently, it is one of the most widely used drugs in the clinical treatment of CVS.^17^-^19^ In addition, alprostadil is a Ca^2+^ antagonist. Its injection is carried by lipid microsphere, which not only ensures drug activity, but also helps it target the site of vascular damage.^20^,^21^

Plasma viscosity, whole blood viscosity at high shear, whole blood viscosity at low shear, hematocrit and platelet adhesion are important indicators of blood fluidity. The higher the values, the higher the blood viscosity and the resistance during blood flow, which will affect the blood supply of organs, thus leading to diseases. Our results demonstrated that compared with the control group, the above hemorheological indexes in the observation group decreased to lower levels, suggesting that the combined treatment using alprostadil and nimodipine can improve blood viscosity, restore microcirculatory perfusion, and is conducive to the stability and improvement of the disease. This may be caused by that as a prostaglandin, alprostadil is easy to bind to prostaglandin receptor of vascular smooth muscle, which is of great significance for vasodilation and platelet aggregation. It can target to improve cerebral vascular microcirculation and alleviate spasm.^22^,^23^

### Limitations:

It includes small sample size, and only preliminary exploration on the clinical efficacy of alprostadil combined with nimodipine in elderly patients with CVS. We plan to further analyze the specific mechanism of the combination of the two drugs in the treatment of CVS patients.

## CONCLUSION

Alprostadil combined with nimodipine can significantly reduce serum inflammatory factor levels and hemorheological indexes, alleviate clinical symptoms, restore microcirculatory perfusion and improve efficacy in elderly patients with CVS after SAH.

### Authors’ Contributions:

**SL and NG** designed this study, prepared this manuscript, are responsible and accountable for the accuracy or integrity of the work.

**JX and YZ** collected and analyzed clinical data.

**FS and TJ:** Data analysis, significantly revised this manuscript.
